# Recent Advances in Electrochemical Sensors for the Detection of Biomolecules and Whole Cells

**DOI:** 10.3390/biomedicines9010015

**Published:** 2020-12-26

**Authors:** Intan Rosalina Suhito, Kyeong-Mo Koo, Tae-Hyung Kim

**Affiliations:** 1 School of Integrative Engineering, Chung-Ang University, Seoul 06974, Korea; intanrosalinasuhito@gmail.com (I.R.S.); sse0913@naver.com (K.-M.K.); 2 Integrative Research Center for Two-Dimensional Functional Materials, Institute of Interdisciplinary Convergence Research, Chung Ang University, Seoul 06974, Korea

**Keywords:** electrochemical method, nanobiosensors, biomolecules, stem cells, cancer cell detection

## Abstract

Electrochemical sensors are considered an auspicious tool to detect biomolecules (e.g., DNA, proteins, and lipids), which are valuable sources for the early diagnosis of diseases and disorders. Advances in electrochemical sensing platforms have enabled the development of a new type of biosensor, enabling label-free, non-destructive detection of viability, function, and the genetic signature of whole cells. Numerous studies have attempted to enhance both the sensitivity and selectivity of electrochemical sensors, which are the most critical parameters for assessing sensor performance. Various nanomaterials, including metal nanoparticles, carbon nanotubes, graphene and its derivatives, and metal oxide nanoparticles, have been used to improve the electrical conductivity and electrocatalytic properties of working electrodes, increasing sensor sensitivity. Further modifications have been implemented to advance sensor platform selectivity and biocompatibility using biomaterials such as antibodies, aptamers, extracellular matrix (ECM) proteins, and peptide composites. This paper summarizes recent electrochemical sensors designed to detect target biomolecules and animal cells (cancer cells and stem cells). We hope that this review will inspire researchers to increase their efforts to accelerate biosensor progress—enabling a prosperous future in regenerative medicine and the biomedical industry.

## 1. Introduction

There is a pressing need for rapid clinical monitoring and diagnostic approaches that associate high sensitivity, selectivity, and quick performance with sample determination [1]. Recent progress in bioanalytical techniques has led to integrating conventional biological concepts with digital instrumentation to establish an easy-to-use, handheld system [2,3]. Biosensors are a significant breakthrough in scientific research, which could be defined as the device that relies on specific biochemical reactions involving isolated enzymes, immune systems, tissues, organelles or whole cells during the electrical, thermal, or optical signals detection of chemical compounds [4]. They promise to enable the detection of biologically related substances through biorecognition and signal transduction cost-effectively, highly accurately, and rapidly [5,6]. Several researchers have attempted to develop a sensor device that can quickly detect antibodies, antigens, enzymes, proteins, and DNA in complex samples [7,8,9]. Following those innovations, biosensing entities have received significant attention for changing the medical paradigm from treatment to prevention and diagnoses due to the potential of biomolecules as disease biomarkers [10,11].

Many types of biosensor modes have been developed based on physicochemical signal transducers, such as electrochemical or fluorescence phenomena, surface plasmon resonance (SPR), and field-effect transistor (FET) [12,13,14,15]. An electrochemical biosensor is defined as a reliable integrated system that serves quantitative or semi-quantitative analytical profiles from a target of interest through interactions between biochemical receptors and electrochemical transducer elements [16,17,18,19]. For electrochemical sensing-based biosensors, the detection process is less expensive and more rapid than conventional methods [20,21,22]. Furthermore, it has promising performance for point-of-care (POC) in label-free operation and simple miniaturization. Numerous studies have reported an increase in biosensing platform sensitivity and selectivity, which indicates that lowering the sensor detection limit toward specific target molecules is possible [23,24,25,26].

Further architecture modifications have been performed with materials that enhance surface conductivity to increase sensor sensitivity and selectivity [27]. Nanomaterials have been essential in reinforcing various constituents that have eventually become biosensing tools [28,29]. Metal nanoparticle-based electrochemical biosensors have been intensively studied due to evidence that the biological or chemical reactions between biomolecules are more accessible due to their interactions with metal structures [30,31].

Other than metal nanoparticles, carbon-based materials have also become highly attractive in the development of an electrochemical sensing platform (e.g., graphene and its derivatives and carbon nanotubes), mostly due to their favorable characteristics such as excellent performance, high mechanical strength, and thermal stability [32,33,34]. Moreover, peptide molecules and oligonucleotides—called aptamers—have been developed, which have received significant attention in biosensor modification strategies because they enable high-affinity binding to a specific target molecule and can convert biorecognition cues into electrochemical signals [35].

With the ability of electrochemical sensing to operate non-destructively and non-invasively, a direct in situ detection method is considered as an analytical tool for the living system to underrate the use of chemical agents such as chemical dyes, radio-labeling, and fluorogenic probes [36,37,38]. Some studies have reported the potential of electrochemical detection to access highly proliferative cell viability (e.g., cancer cells and pluripotent stem cells) through their cell-redox properties, leading to the advancement of whole-cell sensing [39,40,41,42,43]. Through electrochemical biosensing, toxicity assessment and early diagnosis can be efficiently conducted without any adverse effects on live cells [44,45,46,47]. Hence, this is widely applicable according to their advantages, such as in drug screening, stem cell pluripotency monitoring, and differentiation [48,49].

**Figure 1:**
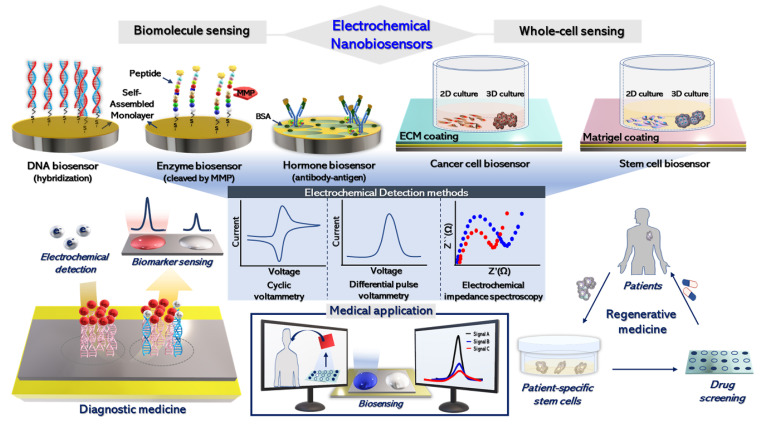
Studies of electrochemical sensors for various target molecules and cells.

In this review, we describe the progress in electrochemical sensors for detecting targets of interest, from biomolecules to the cellular level corresponding with cell viability toward cancer cells and pluripotent stem cells (PSCs) as highly proliferative cells (Figure 1). Furthermore, we comprehensively discuss various nanomaterials and molecules, combined with biosensing platforms, that increase electrochemical sensitivity and selectivity. Accelerating the progress of biosensing technology will require attention for future studies in biomedicine and regenerative therapies.

## 2. Electrochemical Detection of Biomolecules

The detection of small biomolecules (e.g., nucleic acids, enzymes, and hormones) is based on their biological and physiological functions: transmitting genetic information, regulating biological activity, and catalyzing reactions at the cellular level [50,51,52]. However, developing biomolecule sensing technology remains a challenge [53,54]. Standard biomolecular techniques for analyzing biomolecules have been developed, such as gel electrophoresis, Western blot, and polymerase chain reaction (PCR) [55]. Despite precise characterization results, they are hindered by limitations such as expensive reagent requirements, laboriousness, and high time requirements [56]. The electrochemical detection method has significant potential to address the drawbacks of conventional methods with fast accessibility, cost-effectiveness, and high sensitivity and selectivity toward a specified target.

Numerous papers have reported the studies of electrochemical techniques for detecting small biomolecules as an early diagnosis [26]. The electrochemical detection of DNA and RNA has been used to diagnose viral infections, such as coronavirus, Zika virus, and hepatitis E [57,58,59,60,61]. Another example is electrochemical-based enzyme and hormone detection to inspect cancer, pregnancy, food toxicity, and pollution levels. Compared with conventional methods (Western blot and PCR) that are costly and time-consuming, the electrochemical approach is a superior alternative [62,63]. Nonetheless, its performance is not distinct from its conductivity properties [64,65,66]. Furthermore, the signal-overlapping from interference must be hindered for electrochemical performance with complex samples [67]. This section presents representative examples of electrochemical sensing platforms for nucleic acids, enzymes, and hormones, as summarized in Table 1.

### 2.1. Electrochemical DNA Sensing Platforms

**Figure 2:**
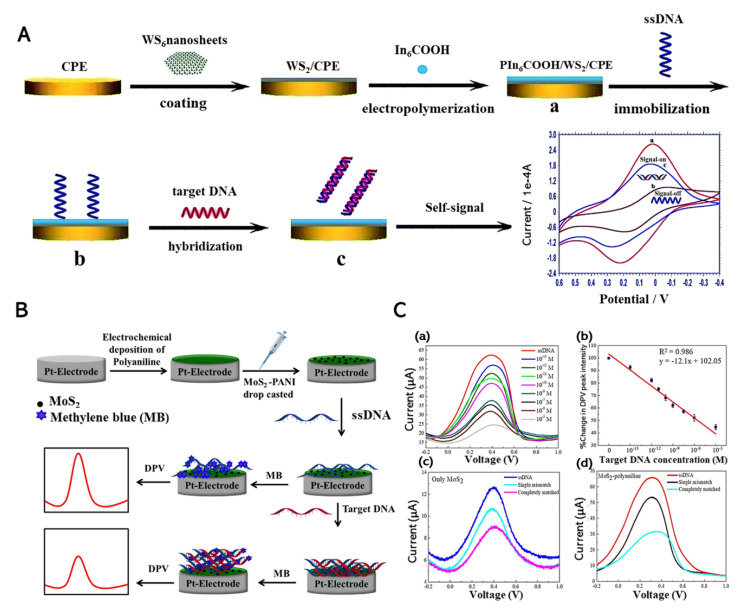
(**A**) Construction process for a self-signal electrochemical sensing platform. (**B**) Fabrication of the electrochemical sensors for DNA hybridization and quantitative DPV detection. (**C**) DPV graphs obtained for (**a**) Pt||MoS_2_-polyaniline-ssDNA electrode with different target DNA concentrations, (**b**) calibration curve of DNA detection in term of percentage change in DPV intensity for target DNA concentration, (**c**) DNA detection of bare Pt||MoS_2_-ssDNA electrode, and (**d**) Pt||MoS_2_-polyaniline-ssDNA electrode of single-mismatched and fully matched DNA. Reprinted with permission from [69]. Copyright 2019, Royal Society of Chemistry; Represented with permission from [70]. Copyright 2018, Elsevier.

Electrochemical genosensing technology for DNA diagnostics has been recently developed [68,83]. Of the DNA sensing methods, electrochemical detection has the advantages of inexpensive equipment, sensitivity, and rapid performance. Compared with sequence-specific DNA detection, genosensing is a promising biosensor technology, particularly for early disease diagnosis, forensic application, and drug screening [84,85]. Immobilization performed in several steps is vital to the electrochemical detection of DNA. According to Yang et al. (2019), self-signal DNA detection was performed via the immobilization of hybridized ssDNA [69]. Firstly, tungsten disulfide (WS_2_) nanosheets were treated on a carbon paste electrode (CPE), followed by poly (indole-6-carboxylic acid) (PIn_6_COOH) treatment. Consequently, a WS_2_/PIn_6_COOH nanocomposite was formed, and an ssDNA probe was attached to the WS_2_/PIn_6_COOH nanocomposite-modified CPE through the redox response. The electrode was then immersed with a 1.0 × 10^−11^ mol L^−1^ ssDNA probe containing 10 mL of phosphate-buffered solution (PBS, pH 7.0). After immersing the electrode, the ssDNA probe was non-covalently assembled on the WS_2_/PIn_6_COOH nanocomposite. Altogether, the DNA was immobilized through the hybridization step (Figure 2A). Cyclic voltammetry (CV) and electrochemical impedance spectroscopy (EIS) were used prior to the detection of DNA immobilized on the CPE electrode [86]. The ssDNA probe was hybridized with the target DNA to form the double-helix structure, which induced the dsDNA release from the nanocomposite surface.

Dutta et al. (2018) proposed a platform that could successfully detect concentrations as low as 10^−15^ M of target DNA without labeling or amplifiers (Figure 2B) [70]. The Pt||MoS_2_-polyaniline electrode is immersed in a tris buffer (pH 6.9) containing 2.5 μM ssDNA solution, which functions as a DNA probe. This reported DNA biosensor directly senses the target through differential pulse voltammetry (DPV) and CV measurements with a wide linear range of detection (10^−15^ to 10^−6^ M). The electrochemical characteristics of methylene blue (MB) on an ssDNA/MoS_2_-polyaniline/Pt electrode was conducted in different conditions (Figure 2C). The sensor treated with a MoS_2_-polyaniline nanocomposite successfully detected the target DNA in various target DNA solution concentrations, as depicted in Figure 2C(a).

Furthermore, a DPV measurement was conducted with a MoS_2_-polyaniline electrode and an only-MoS_2_ electrode (Figure 2C(c,d)). The peak current of completely matched DNA was diminished compared with that of single-matched DNA. DNA sensor detection could occur at an extremely low concentration (down to 10^−15^ M) using the electrochemical method, a superior alternative to conventional methods. Furthermore, the novelty of DNA-sensing technology primarily concerns the hybridization of target DNA on conductive platforms without labeling or pretreatment. In the future, further improvement is required for more accurate DNA recognition and detection.

### 2.2. Electrochemical Biosensors for Enzyme Activity

Enzymes are essential proteins in the body due to their fundamental role in the four stages of degradation, absorption, oxidation, and reduction [87]. Enzymes can be found throughout the body because they flow in the blood and enter cells in each organ [88,89]. Many researchers attempt to construct platforms that can recognize any specific enzyme that could function as a biomarker without causing damage. The electrical signal is based on enzymes’ actions in activating and degrading specific substances. For example, ferrocene and potassium ferricyanide are used to indirectly confirm enzyme activity, and fluorescent substances are labeled to develop a sensing platform. Recent studies have successfully achieved a high limit of detection (LOD), which is highly favorable for further quantitative approaches in biomedicine and diagnostics. In contrast, confirming the capability of the electrochemical method for the qualitative characterization of enzyme features is challenging because the features exist in the complex form. In this section, we describe several recently reported sensors that enable enzyme detection, which could be used as a proof-of-concept and motivation for future development.

#### 2.2.1. Electrochemical Detection of Thrombin

Thrombin is a type of serine protease essential to molecular biology for tumor growth, metastasis, angiogenesis, and blood coagulation [90]. It is used primarily as a tumor marker to diagnose pulmonary metastasis [91,92]. High or low blood thrombin levels are associated with blood coagulation [93]. Accordingly, the specific and quantitative detection of thrombin is vital in clinical practice and diagnostic approaches. Recently, thrombin-bound aptamers have successfully demonstrated the capability to use various transducers as molecular receptors [94,95]. Thrombin has been successfully detected using several methods, such as fiber-optics, fluorescence-based, and infrared fluorescence sensors [96,97]. However, these methods lack operation and detection times. Considering blood coagulation and environmental complexity, developing the ability to detect thrombin electrochemically is challenging [71,98].

**Figure 3:**
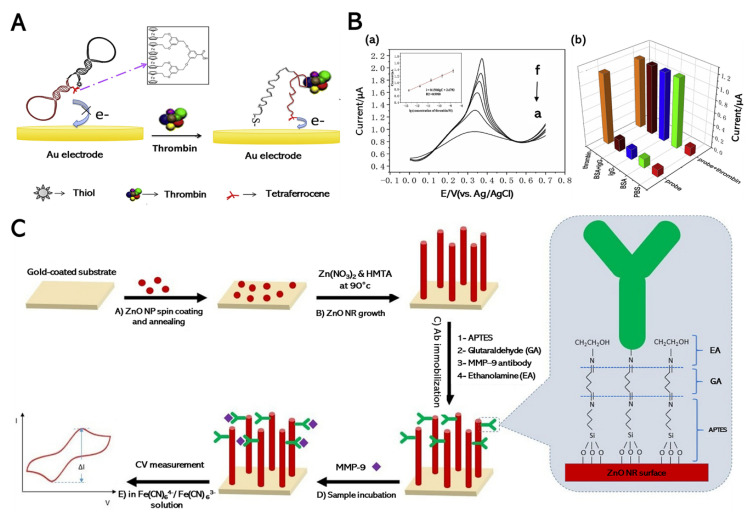
(**A**) Electrochemical sensor for thrombin detection. (**B**) DPV (a) response of different thrombin concentrations, a: 0 M, b: 1.8 × 10^−13^ M, c: 1.8 × 10^−12^ M, d: 1.8 × 10^−11^ M, e: 1.8 × 10^−10^ M, and f: 1.8 × 10^−9^ M target thrombin. (Insert) Calibration plots of target thrombin (1.8 × 10^−13^ to 1.8 × 10^−9^ M). Specificity of the assay for the detection of thrombin (**b**). (**C**) Biosensor for the detection of MMP-9 biomarker. (**A**) Spin-coating and annealing of ZnO nanoparticle seed solution, (**B**) ZnO nanorod growth, (**C**) antibody immobilization, (**D**) sample incubation, and (**E**) electrochemical measurement (CV and EIS). Chemical link between ZnO surface and antibody is illustrated on right side. Reprinted with permission from [72]. Copyright 2020, Elsevier; Reprinted with permission from [75]. Copyright 2020, Elsevier.

According to the platform developed by Cheng et al. (2020), a homogenous electrochemical biosensor based on a selected aptamer probe was fabricated with tetra-ferrocene at the 3′ terminal and a thiol group at the 5′ terminal for sensing thrombin, as described in Figure 3A [72]. This reported biosensor successfully enhanced the binding efficiency between the target unit and substrate, including a wide range of concentrations, in the range of 0.18 to 1.8 nM (Figure 3B(a)). Various common proteins, such as Immunoglobulin G (IgG) and bovine serum albumin (BSA), were used to confirm the specificity of this reported sensor; the thrombin probe did not selectively interact with either protein (Figure 3B(b)). Furthermore, Zhang et al. (2018) proposed a platform where two aptamers are directly used as the recognition unit and electroactive indicator to detect thrombin [73]. The graphene promotes the electron transfer and amplifies the electrochemical signals [99,100]. The detection limit is 0.03 fM, which indicates the proposed electrode’s high sensitivity level. For selectivity, the reported biosensors demonstrated that only thrombin significantly increases the signal compared with other serum samples (e.g., lysozyme and BSA). The developed platform is promising for highly sensitive and selective thrombin detection.

#### 2.2.2. Electrochemical Detection of Matrix Metalloproteinase

Matrix metalloproteinase (MMP) is a zinc-dependent proteolytic enzyme capable of degrading all components of the extracellular matrix (ECM) [101]. It consists of 24 types that differ preferentially based on the enzyme substrates: type IV collagenase (MMP-2 and MMP-9), stromelysin (MMP-3), and interstitial collagenase (MMP-1) [102,103]. MMP has properties involved in tumor invasion and metastasis and functions as a biomarker for infection, inflammation, and cancer growth [104]. Consequently, it is essential to precisely detect its activities and quantities at the cellular level [74]. Lee et al. (2016) reported that the redox reporter MB labeled with the peptide was used to integrate a gold-based biosensor [76]. Classical lithography and etching processes are defined as production techniques where the working electrode can maintain a continuous potential without the reference electrode [105]. This simplified platform is further applicable in electrochemical-based cancer diagnosis.

Shabani et al. (2020) reported MMP-9 detection by the electrochemical method using zinc oxide (ZnO) nanoparticles and a ZnO nanorod-modified substrate [75]. An Au-coated substrate was modified using ZnO nanoparticles, and its concentration was optimized using the CV method. Antibody immobilization was then performed by (3-aminopropyl)triethoxysilane (APTES), glutaraldehyde, and ethanolamine treatment for high platform sensitivity and selectivity (Figure 3C). Furthermore, ZnO nanorods were fabricated using the hydrothermal method: a ZnO nanoparticle seed layer was coated on the Au-coated substrate, followed by MMP-9 substrate conjugation via antibody immobilization. Based on the CV graph, MMP-9 was detectable at a concentration of 1 to 1000 ng mL^−1^ with a detection limit of 0.15 ng mL^−1^. Similar to the commercial enzyme-linked immunosorbent assay (ELISA) in real serum samples, the mean MMP-9 concentration was detected by the CV methods. Remarkably, this proposed biosensor achieved a lower detection limit of 7% than commercial ELISA at 10%. The reported studies confirmed the efficiency of direct MMP detection through its electrochemical behaviors, which will be of further use before its application in POC diagnosis.

## 3. Electrochemical Biosensors for Hormone Detection

The development of biosensors enables detecting biomolecules and other phenomena, including hormones [106,107]. Hormones are secreted primarily by glands or specific cells, circulate in the bloodstream, and specialize in targeting cells [108]. The electrochemical biosensing of hormones has emerged for treating human diseases and performing clinical diagnosis. The quantity of hormones that regulate and control the metabolism of the human body is very low, leading to efforts to develop a highly sensitive tool to detect them. The electrochemical approach has typically been used for hormone sensing because it can overcome the limitations of other well-established methods (e.g., ELISA) in terms of sensitivity, selectivity, and time performance [109,110]. One example is the modified screen-printed carbon electrode (SPCE) with cobalt nanoparticles (CoNPs) with chitosan and multi walled carbon nanotubes (MWCNTs) (CoNPs/chitosan-MWCNTs/SPCE) that can successfully detect insulin with concentrations down to 25 nM. This finding could confirm the advantages of an electrochemical detection system [111]. This section describes several current sensors for hormone detection that may contribute to the development of electrochemical-based hormone sensors.

### 3.1. Electrochemical Detection of Estrogen Hormone

Estrogen is a naturally occurring steroid hormone in mammals with unusual behavior when it reacts with its receptor [78]. It is an essential hormone in the female reproductive cycle, menstrual cycle, and growth, while it can also lead to obesity and infertility at abnormal levels. Eighty percent of breast cancers are affected by estrogen, indicating its association with cancer [112]. Therefore, estrogen-level detection is highly favorable due to its promotion effects and initial tumor formation. A detection platform that uses the estrogen receptor has been studied through an electrochemical detection platform that is non-destructive with high selectivity and sensitivity [77,113,114]. In 2018, Liu et al. developed an electrode surface transformation with a gold electrode on which 6-mercapto-1-hexanol (MCH) was used for a self-assembled monolayer (SAM) [79]. Graphene was treated with a bi-function to adsorb the E2-binding aptamers and the SAM on the MCH/Au modified electrode. Electrochemical detection was performed with a 20 mM PBS containing 5 mM FcCOOH and 0.1 M NaClO_4_. The DPV performance confirmed the enhanced detection of E2.

**Figure 4:**
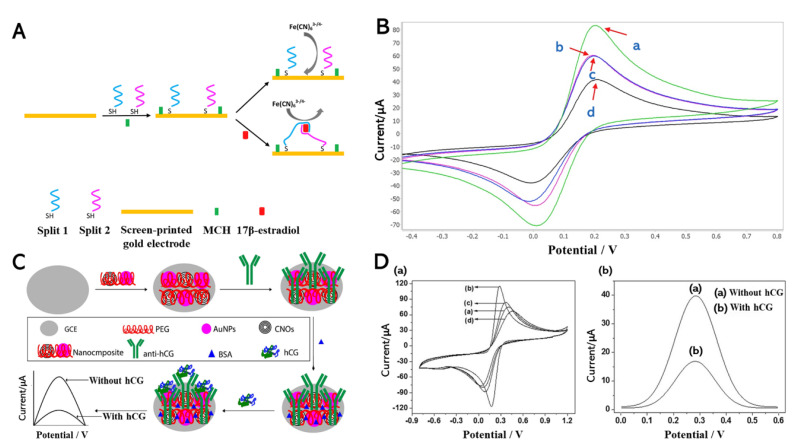
(**A**) Electrochemical aptasensor for sensing 17β-estradiol (E2) based on split DNA aptamers. (**B**) Electrochemical characterization of the electrode modification and the aptasensor function. CV profiles of: bare electrode (green curve, curve a), split DNA aptamers-modified electrode (pink curve, curve b), split DNA aptamers-modified electrode + bisphenol A (BPA) (lack of bridge) (blue curve, curve c), split DNA aptamers-modified electrode + E2 (bridge assembly) (black curve, curve d). (**C**) Fabrication of the hCG-immunosensor. (**D**) Assessment of the step-wise fabrication of hCG-immunosensors and electrochemical signal: (**a**) CV curve of (a) bare-GCE, (b) CNOs/AuNPs/PEG/GCE, (c) anti-hCG/CNOs/AuNPs/PEG/GCE, and (d) BSA/anti-hCG/CNOs/AuNPs/PEG/GCE; SWV curves (**b**) of immunosensor (a) without hCG and (b) with hCG. Reprinted with permission from [78]. Copyright 2019, Elsevier; Reprinted with permission from [81]. Copyright 2019, Elsevier.

Nameghi et al. (2019) used the gold electrode and aptamers for the substrate to detect estrogen (Figure 4A). Previously, aptasensors have demonstrated satisfactory results at detecting their targets [113,114]. The immobilization was performed via thiol-modified split aptamers that can react with the gold surface [78]. CV was conducted through the proposed platform because the interfacial reaction could be determined via this method, thus enabling easy discrimination between estrogen and the control group through their signal (Figure 4B). The bare electrode presented the maximum CV current (curve A), which indicated excellent electron transfer between the bare gold electrode and [Fe(CN)_6_]^3−/4−^. When split aptamers were conjugated onto the gold electrode’s surface, the redox current decreased (curve B). From curve D, when adding the E2, the electrochemical signal was significantly reduced (curve D) because the split1–E2–split2 complex was formed on the electrode. However, in the presence of bisphenol A (BPA) as non-target substances, the current signal of the split DNA aptamer modified electrode did not change (curve C). Furthermore, DPV analysis was performed, in which the concentrations of E2 were measured from 1.2 pM to 100 pM and 100 nM to 7 nM with a detection limit of 0.5 pM (S/N = 3). The outstanding performance of the proposed biosensors demonstrates their reproducibility, high sensitivity, and selectivity.

### 3.2. Electrochemical Detection of Human Chorionic Gonadotropin (hCG) Hormone

Human chorionic gonadotropin (hCG) is a glycoprotein hormone secreted by placenta trophoblast cells that functions as a diagnostic marker for pregnancy and a tumor marker [115]. The early quantitative detection of hCG is particularly challenging. Numerous hCG analysis methods have been reported, such as ELISA, fluorescence-labeled immunoassay, and radioimmunoassay [116,117]. Electrochemical detection could overcome the limitations of other methods (e.g., high cost, laborious, slow performance, and risk potential of radioactivity) [80]. A highly sensitive electrochemical immunosensor based on carbon nano-onions (CNOs), gold nanoparticles (GNPs), a polyethylene glycol (PEG) composite, and a glassy carbon electrode (GCE) was reported by Rizwan et al. (2019) [81]. This composite was drop-casted onto a pre-cleaned GCE as a self-assembled monolayer via chemisorption. The anti-hCG was then immobilized onto the modified CNOs/AuNPs/PEG/GCE biocompatible interface (Figure 4C). Before detecting the hCG, this fabricated sensor was incubated for 45 min at room temperature. This layer-by-layer fabrication was conducted through CV characterization. The detection of hCG was performed using square wave voltammetry (SWV), as illustrated in Figure 4D. This hCG immunosensor exhibited high sensitivity and productivity at a low detection concentration of 100 fg/mL.

Damiati et al. (2019) developed a screen-printed sensor based on the modified carbon macro- and micro-electrodes with a linker, 1-pyrenebutyric acid-N-hydroxy-succinimide ester (PANHS), and the immobilization of anti-hCG antibodies to detect hCG [82]. CV was conducted to characterize the modified electrode by increasing the scan rate from 10 to 100 mV/s. Furthermore, the SWV detection of the micro-electrode exhibited a higher sensitivity (1 pg/mL) than the macro-electrode sensor with a lower detection limit of 100 pg/mL. The working electrode’s physical size directly impacted the electrochemical sensitivity of biosensors that used macro- and micro-electrodes. The results of CV and SWV performance on the modified BSA/anti-hCG antibody/PANHS/SPCE demonstrated that the low-cost, label-free biosensor has high selectivity for hCG detection.

## 4. Electrochemical Biosensing for Highly Proliferative Cells

In addition to detecting biomolecules, it is essential to sense larger-unit cells, useful for prophylactic and therapeutic tools in disease modeling [118,119]. Sensing technology at the cellular level is challenging compared with biomolecule detection [29,45] due to its complex structure and composition, including a sensitive microenvironment that varies depending on the culture conditions, temperature, pH, and nutrient supply [120]. Several classical methods, such as immunostaining, PCR, and flow cytometry analysis (FACS), are commonly employed in cell and tissue characterization even though living samples become irretrievable after such analyses. A cell-based biosensor is a promising solution for sensitive, reliable, and non-destructive cell viability measurement [45]. Numerous studies of electrochemical-based biosensors for disease modeling and diagnostics have been reported [121,122,123]. For instance, electrochemical biosensors of cell cycles and growth factors highly favorable for cancer treatment have been established [124,125].

In contrast, electrochemical biosensors have emerged as an innovative method for stem cell live sensing [126,127,128]. The ability to monitor stem cell pluripotency and differentiation rapidly and non-destructively is useful [129,130]. For example, electrochemical biosensing has been used to assess osteogenesis and neurogenesis of stem cells as therapeutic agents in regenerative medicine [131,132,133]. For this advanced study, high sensitivity, selectivity, and ease of handling the stem cell culture are the primary concerns for further improvement. Moreover, cell activity and metabolism can be electrically monitored for living cells, yet the handling system still depends on a laboratory scale [134,135].

Accordingly, the continuous research and improvement of electrochemical-based technology are necessary to upscale current electrochemical systems to commercialize stem cell-based products [136,137,138]. Biosensing platforms are usually combined with non-biomaterials, including nanoparticles, due to the cell–substrate interactions that may enhance the readable signal transduction. This section summarizes the development of electrochemical biosensors for cancer cells and stem cells as typical, highly proliferative cells (Table 2).

### 4.1. Electrochemical Detection of Cancer Cell Viability

Before cancer prevention and treatment, detecting cancer cells at an early stage by sensing their presence in the human body is essential [149]. In vitro cancer-cell detection based on the electrochemical method—which provides label-free, non-invasive, and non-destructive performance that could further support anticancer drug discovery—has emerged recently [120,139,140,141,142,143,150,151]. Angeline et al. (2020) reported the electrical signal enhancement of stomach cancer cell (MKN-28) viability through the electrochemical detection method, which is then useful for drug screening applications [144]. The ECM-coated hybrid platform was optimized for the electrochemical assessment of MKN-28 cells, followed by treatment with anticancer drugs and cell viability assessment. The signal was enhanced using ECM coating before the cell culture was placed on the ECM-coated hybrid platform, indicating the ability of ECM to accelerate cell–substrate interaction.

**Figure 5:**
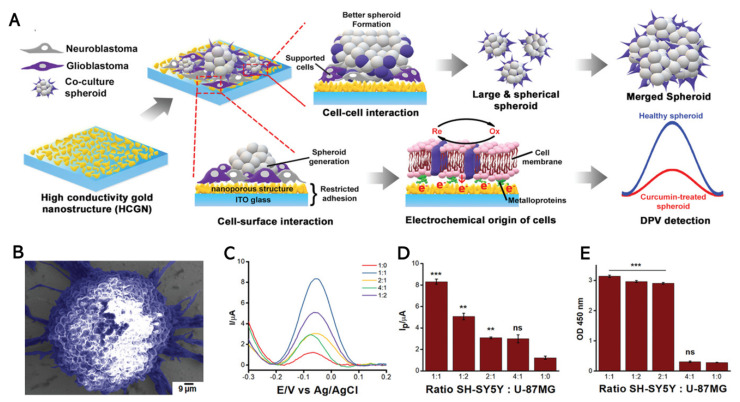
(**A**) Superiority of highly conductive gold nanostructures (HCGN) platform for spheroid formation and electrochemical detection using DPV. Co-cultured spheroids consisting of SH-SY5Y and U-87MG cells were more effective at mimicking the in vivo conditions of brain tumors than a 2D monolayer culture of co-cultured cells, and the bio-multifunctional sensor containing spheroids could be generated in conjunction with HCGN. (**B**) Pseudo-colored SEM images of co-cultured SH-SY5Y and U-87MG cells (1:1 ratio) and spheroid structure on modified substrates on day 7 of in vitro culture. (**C**) DPV signals from spheroids generated with different cell ratios. (**D**) Calculated peak intensities observed in (C) are presented as a bar graph (I_p_ represents the intensity of the peak current, Student’s *t*-test, *N* = 3, ** *p* < 0.01, *** *p* < 0.001). (**E**) CCK-8 results from spheroids generated with different cell ratios (Student’s *t*-test, *N* = 3, *** *p* < 0.001). Reprinted with permission from [145]. Copyright 2020, Wiley Online Library.

Recently, Suhito et al. (2020) proposed a bio-multifunctional platform that can simultaneously perform 3D multicellular cancer spheroid formation and real-time assessment of the anticancer drug treatment (Figure 5A) [145]. The indium tin oxide (ITO) glass electrode was modified with HAuCl_4_ via electrodeposition, as previously reported. This platform consists of highly conductive gold nanostructures (HCGN) that enable the spontaneous formation of spheroids and detect their viability using the electrochemical method. The surface roughness of gold nanostructures reduces cell adhesion and thus supports automatic spheroid formation. Furthermore, gold nanoparticles have high conductivity, long-term stability, and high biocompatibility favorable for their implementation in electrochemical detection.

DPV was performed toward co-culture spheroid formation in multiple ratios of SH-SY5Y and U87-MG cells. Based on Figure 5B,C, the 1:1 ratio was the most preferable for the electrochemical method and spheroid formation. However, the colorimetric method (CCK-8 assay) results exhibited no significant differences of co-culture spheroid viability with ratios of 1:1, 1:2, or 2:1, which suggests that electrochemical detection has higher sensitivity than conventional analysis (Figure 5D,E). Furthermore, this co-culture spheroid system on a multifunctional platform has been used for anticancer drug screening. The platform can detect toxicity of a low concentration of curcumin (70 µM) after 35 h of incubation and a high concentration of curcumin (500 µM) within a short amount of time (<7 h), which is incredibly difficult to discern using conventional colorimetric methods. Therefore, this platform is highly promising as a label-free high-throughput drug screening method for 3D cell culture systems.

### 4.2. Electrochemical Sensing of Stem Cell Pluripotency

Stem cells keep pace with the rapid development of high-precision medical technology, use the genetic information of individuals to create the same tissue with the in vivo environment, and are derived from the patient’s tissue [152,153,154,155]. They exist in various types, such as mesenchymal stem cell (MSC), embryonic stem cell (ESC), and neural stem cell (NSC), which could further be differentiated into specific cells such as neurons, adipocytes, oligodendrocytes, osteoblasts, and chondrocytes [156]. During the development of drugs, stem cell differentiation is exceptionally favorable for obtaining specific cells of interest. These cells are further treated with drug candidates to confirm drug safety and efficacy [157,158]. Many recent studies have reported the non-destructive and label-free detection of stem cell pluripotency and differentiation via electrochemical biosensors to avoid irreversible cell damage after analysis [146,159,160,161].

Human embryonic stem cells (hESCs) are categorized as PSCs capable of differentiating into specific cells and could constitute more than 210 human organs, which can be widely used to treat intractable diseases. Nevertheless, the formation of teratoma due to the undifferentiated state of PSCs is a serious problem in clinical applications. Thus, it is vital to develop methods that can enable the precise characterization and screening of PSCs without destroying or damaging the differentiated cells. Jeong et al. (2016) proposed a modified hybrid film that consists of an arginyl-glycyl-aspartic acid (RGD)-MAP-C peptide, gold nanoparticle (GNP) film, and a Matrigel layer, which manifests the electrochemical detection of undifferentiated hESCs [147]. This platform exhibited detection ability with as few as 25,000 hESCs, which was 2.8 times more sensitive than in previous research. Furthermore, hESC-derived MSCs were subjected to DPV detection to confirm this platform’s sensitivity toward hPSCs. The MSC electrical signals were negligible compared with the DPV signal of undifferentiated hESCs.

**Figure 6:**
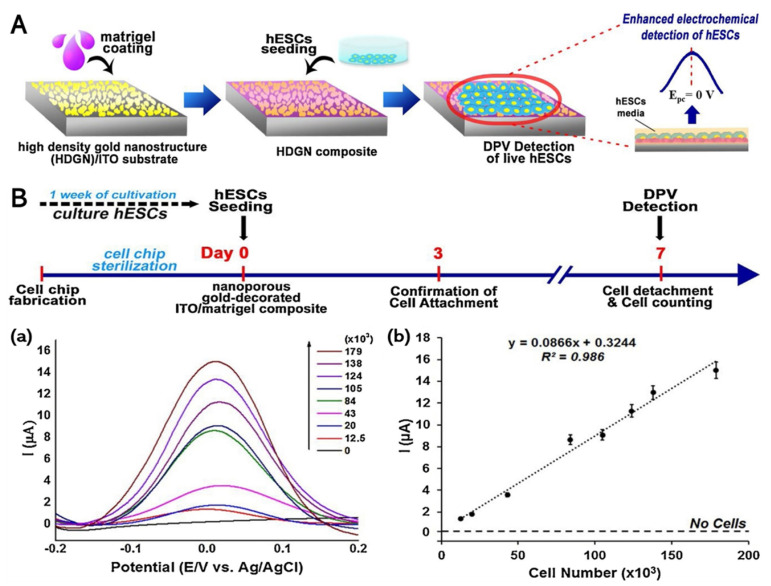
(**A**) Optimization of a high-density gold nanostructure (HDGN) composite for the enhancement of human embryonic stem cells (hESC) electrochemical signals; (**B**) (**a**) DPV signals achieved using various hESC quantities in the range of 12,500–179,000 cells, presented in the form of an XY graph. (**b**) Linear correlations (R^2^) between the calculated I_p_ value from DPV signals and the number of hESCs. Reprinted with permission from [148]. Copyright 2019, Elsevier.

Given the possibility of teratoma formation from the marginal number of hPSCs, further optimizations are needed to significantly improve biosensor sensitivity. Accordingly, Suhito et al. (2019) developed an hPSC-sensing platform by optimizing the Au-film structure formation through the electrochemical deposition process, which further accelerated the sensitivity and selectivity toward hESCs (Figure 6A); the composite was called a high-density gold nanostructure (HDGN). This complex enhances the redox signal in living cells, indicating that the cell adhesion and growth were functionally increased [148].

The LOD was significantly increased (by twofold) with our developed platform compared with the RGD–MAP–C on the Matrigel-coated GNP film—approximately 12,500 hESCs were successfully detected by DPV measurement (Figure 6B). Furthermore, it exhibited high selectivity toward hESCs in the presence of human cord blood-endothelial progenitor cells (hCB-EPCs) as a normal cell (40,000 cells/chip). Hence, this platform is promising for biosensors used in stem cell applications for tissue regeneration and clinical therapy.

## 5. Conclusions and Future Perspectives

This paper summarizes the successful electrochemical sensors that have been designed to detect small biomolecules (e.g., DNA, enzymes, and hormones) and the complex of cells. The electrochemical method is a rapid, precise, and non-destructive tool to analyze a broad range of targets of interest. Functional peptides, aptamers, and nanomaterials (e.g., metal nanoparticles, graphene, and graphene derivatives) have been used to increase sensitivity. The interactions between targets and specific probes or composites generate a detectable read-out signal during electrochemical measurement. Accordingly, methods are considered to develop electrochemical platforms that can sense live cells precisely and rapidly before diagnosis and drug discovery. The possibility of monitoring highly proliferative cells through electrochemical devices has been confirmed, including detecting cancer cell viability and monitoring stem cell pluripotency and differentiation status upon their redox behaviors.

In the future, biosensing technology could contribute toward a useful cell-friendly analysis technique for precision medical diagnosis and POC. Electrochemical sensor technology is an advanced development in biological research. The detection of various substances is feasible, from small biomolecules (e.g., DNA and proteins, enzymes, and hormones) up to the cellular level, corresponding to cell viability. Moreover, it has advantages in sensitivity, selectivity, and processing time, which will be beneficial in future industries. Accordingly, rapid, non-destructive, and applicable electrochemical sensors could be incorporated in sophisticated large-scale systems for disease diagnosis and the quality assurance of stem cell-based products.

## Figures and Tables

**Table 1:** Electrochemical biosensing platforms for detecting biomolecules.

Target	Substrate	Immobilization Strategies	Detection Methods	Ref.
DNA	Screen-printed electrode	Au nanoparticles/TFO probe/Methylene Blue/Target DNA (ssDNA or ds DNA)	CV/SWV	[68]
DNA	Carbon paste electrode	WS_2_/PIn_6_COOH/ssDNA	CV/EIS	[69]
DNA	Platinum electrode	MoS_2_-polyaniline/ssDNA/Methylene Blue (MB)	CV/DPV	[70]
Thrombin	Au electrode	Poly-adenine/aptamer1/thrombin/aptamer2/padlock	CV/DPV/EIS	[71]
Thrombin	Au electrode	Thiol-group/aptamer/tetra-ferrocene	DPV/EIS	[72]
Thrombin	Glassy-carbon electrode	Graphene oxide/MNP-TBA1 (Magnetic nanoparticle-thrombin binding aptamer)/HAP-TBA2 (Hydroxyapatite-TBA2)	CV/SWV	[73]
MMP-2	Au electrode	Selenium/peptide/Na_2_MoO_4_/ssDNA	CV/EIS	[74]
MMP-9	ZnO nanoparticle electrode	Gold-coated glass/ZnO-NP/APTES/Glutaraldehyde/MMP-9 Antibody	CV/EIS	[75]
MMP-9	Au electrode	L-cysteine/EDC/NHS/Peptide/MB	CV	[76]
Estrogen (ER alpha)	Screen-printed electrode	5′-thiol-modified DNA aptamer/Tris-(2-carboxyethyl) phosphine hydrochloride	DPV	[77]
Estrogen (17-β Estradiol)	Au electrode	Split aptamer 1/E2/Split aptamer 2	CV/DPV	[78]
Estrogen (17-β Estradiol)	Au electrode	6-mercapto-1-hexanol (MCH)/Aptamer-Graphene	DPV/EIS	[79]
Human chorionic gonadotrophin (hCG)	Screen-printed carbon electrode	Carbon-nanotube/Antibody 1/hCG/Au-Antibody 2	CV/DPV	[80]
Human chorionic gonadotrophin (hCG)	Glassy-carbon electrode	Carbon nano-onions (CNOs)/gold nanoparticles (AuNPs)/Polyethylene glycol (PEG)	CV/SWV	[81]
Human chorionic gonadotrophin (hCG)	Screen-printed carbon electrode	PANHS/Anti-hCG antibody/BSA/hCG	CV/SWV	[82]

Abbreviations: TFO: triplex forming oligonucleotides, CV: cyclic voltammetry, SWV: square wave voltammetry, EIS: electrochemical impedance spectroscopy, DPV: differential pulse voltammetry.

**Table 2:** Electrochemical biosensing platforms for highly proliferative cells.

Target	Substrate	Immobilization Strategies	Detection Methods	Ref.
MDA-MB-231 cells	Glassy-carbon electrode	Mannose-C_2_NH_2_/Con A or BSA Mannose-C_2_NH_2_/Cell mixture_‘_	CV/EIS	[139]
MCF-7 cells	Glassy-carbon electrode	MWCNT/PGA composite/MUC-1 aptamer/Glutathione/Apt-AgNPs	CV/DPV/EIS	[140]
HepG2 cells	Screen-printed gold electrode	DNA nanotetrahedron/TLS11a aptamer probe/Pd-Pt nanocage (labeled with cDNA)	DPV	[141]
HepG2 cells	Glassy-carbon electrode	Fe_3_O_4_/MnO_2_/Au-Pd/HRP–aptamer/Hemin/G-quadruplex (nano probe)	CV/DPV	[142]
U-87 MG cells	ITO glass electrode	Gold layer/L-cysteine/TAT and RGD-C-peptide	CV/EIS	[143]
HER2 cells	Fluorine doped tin oxide (FTO) glass	Nitrogen-doped graphene/AgNP/Poly aniline/Anti-HER2	DPV	[120]
MKN-28 cells	ITO glass electrode	HAuCl_4_/Fibronectin and Collagen-solution	DPV/EIS	[144]
SH-SY5Y/U-87 MG cells	ITO glass electrode	Gold nanostructure	DPV	[145]
hMSCs	ITO glass electrode	Nano-porous Alumina Mask/Au dot/L-cysteine/RGD-peptide composite	CV	[146]
hESCs	ITO glass electrode	Matrigel/GNPs/RGD peptide/Gold layer	DPV	[147]
hESCs	ITO glass electrode	Matrigel/High density gold nanostructure	DPV	[148]

Abbreviations: ITO: indium tin oxide.
